# An Unusual Cause of Inguinal Mass in a Patient with Urolithiasis: A Case Report of Deep (Aggressive) Angiomyxoma in a Male Patient

**DOI:** 10.3390/clinpract14060213

**Published:** 2024-12-13

**Authors:** Christodoulos Chatzigrigoriadis, Vasileios Tatanis, Theodoros Spinos, Angelis Peteinaris, Angelos Samaras, Anastasios Thanos, Evangelos Liatsikos, Panagiotis Kallidonis

**Affiliations:** 1 School of Medicine, University of Patras, 26504 Patras, Greece; xatzhgrhgoriadhw@gmail.com; 2 Department of Urology, University Hospital of Patras, 26504 Patras, Greece; thspinos@otenet.gr (T.S.); peteinarisaggelis@gmail.com (A.P.); agelos.sam@gmail.com (A.S.); liatsikos@yahoo.com (E.L.); pkallidonis@yahoo.com (P.K.); 3 Mutual Health Fund of National Bank of Greece Personel, 11473 Athens, Greece; anastasios.thanos@gmail.com

**Keywords:** deep angiomyxoma, aggressive angiomyxoma, misdiagnosed inguinal hernia, rare case, case report

## Abstract

**Background:** Deep or aggressive angiomyxoma is an uncommon neoplasm of the pelvis. Although deep angiomyxoma is a benign tumor, its tendency to infiltrate soft tissues and reach a large size (typically > 10 cm) indicates aggressive biological behavior. It is usually present in female patients, but there have been recent reports of male-aggressive angiomyxoma. While rare, it is an important consideration in patients with a pelvic mass. The clinical presentation is non-specific; patients are either asymptomatic or present with non-specific complaints, such as dull pain, constipation, and dysuria. It is commonly mistaken for an inguinal hernia, hydrocele, testicular cancer, lipoma, and epididymal cyst in male patients, thus misguiding the management of these cases. Hence, preoperative evaluation with imaging studies (ultrasound, computed tomography, magnetic resonance imaging) and biopsy allows for an accurate diagnosis and treatment. Currently, the standard of treatment is surgical resection of the tumor with free margins. The role of hormone therapy is under investigation for patients with deep angiomyxoma positive for estrogen/progesterone receptors. Regular follow-up is necessary given the high recurrence rate of deep angiomyxoma (9–72%). **Methods:** We present a case of an elderly man who presented with hematuria due to urolithiasis and an asymptomatic inguinal mass mimicking an inguinal hernia. A computed scan (CT) of the abdomen confirmed the presence of the mass, which was removed surgically. **Results:** The pathologic examination of the tumor was consistent with deep angiomyxoma. **Conclusions:** The diagnosis of deep angiomyxoma should always be considered in patients with an inguinal mass to avoid delayed treatment and incomplete surgical excision.

## 1. Introduction

Deep angiomyxoma constitutes a rare neoplasm usually found in women of reproductive age. The female-to-male ratio is approximately 6–7:1 and it is usually located on the left side [1,2]. Common locations include the vulva, pelvis, and perineum of the female genital tract [3,4,5]. Regarding the male population, it is usually located in the scrotum, pelvis, spermatic cord, inguinal area, perineum, and prostate [4,6,7,8]. The clinical presentation is variable with non-specific symptoms, such as painless swelling or dull pain. Consequently, it is commonly mistaken for an inguinal hernia, hydrocele, testicular cancer, lipoma, or epididymal cyst in male patients due to its rarity and non-specific clinical findings [8,9,10,11]. A typical feature of this neoplasm is the ability to reach a large size by infiltrating soft tissues and displacing the visceral organs [3,5,12,13]. The vast majority of cases represent benign tumors, but the risk of local recurrence is unusually high (9–72%) [3,9,11,13]. Despite the promising outcomes of hormonal therapy, wide surgical excision constitutes the gold-standard treatment approach.

In this case report, we present a patient with an inguinal mass that was removed surgically and eventually diagnosed as deep angiomyxoma after a challenging diagnostic process.

## 2. Case Report

A 70-year-old male presented to our department with hematuria due to a kidney stone and a painless inguinal mass. The mass was present for 4–5 months and was slowly growing. His past medical history was notable for hypertension and dyslipidemia. Physical examination revealed a 5–6 cm non-tender inguinal mass in the absence of inguinal lymphadenopathy. The transillumination test and the Valsalva test were inconclusive. The clinical evaluation of the patient was suggestive of an inguinal hernia.

Urine analysis was remarkable for 12–15 white blood cells (WBCs) per High-Power Field (HPF), >100 red blood cells (RBCs) per HPF, specific gravity 1030 g/mL, pH 5, crystals, and the absence of leukocyte esterase and nitrates. Cell blood count was remarkable for 8480 × 10^6^/L WBCs (79.3% neutrophils, 20.7% lymphocytes), 276,000 × 10^6^/L platelets, hemoglobin 10.3 g/dL, and hematocrit 34.2%. Electrolytes were normal (sodium 142 mmol/L and potassium 4.8 mmol/L), serum urea was 46 mg/dL, and serum creatinine was 1.3 mg/dL. Liver function tests revealed aspartate transferase (AST) 45 U/L, alanine transferase (ALT) 43 U/L, and total bilirubin 0.58 mg/dL. All available tumor markers, including the a-fetoprotein (AFP), beta-human chorionic gonadotropin (β-hCG), Lactate Dehydrogenase (LDH), Carcinoembryonic antigen (CEA), carbohydrate antigen 19-9 (CA19-9) or cancer antigen 125 (CA125), were negative preoperatively.

**Figure 1:**
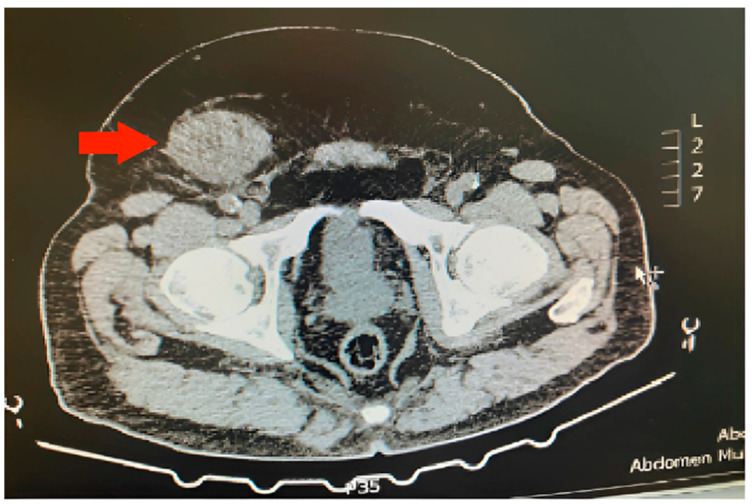
Inguinal deep (aggressive) angiomyxoma (red arrow) on the right side of a male patient.

A computed tomography (CT) scan of the abdomen was ordered for the investigation of urolithiasis, revealing an 8 mm left kidney stone and a right inguinal solid hyperdense mass (Figure 1). The appearance of the inguinal mass was heterogenous with slight contrast enhancement and its size was 6.5 × 5 cm. A decision for surgical excision was taken after informed consent of the patient. The patient underwent an open inguinal incision, and the mass was removed. Intraoperatively, the tumor was encapsulated and it was excised without the rupture of the capsule. Macroscopic invasion of the adjacent organs was not detected. Considering that the excision of the tumor was not complicated, no drain or VAC was placed. This study was conducted in accordance with the Declaration of Helsinki. Considering it is a case report, IRB approval was waived.

**Figure 2:**
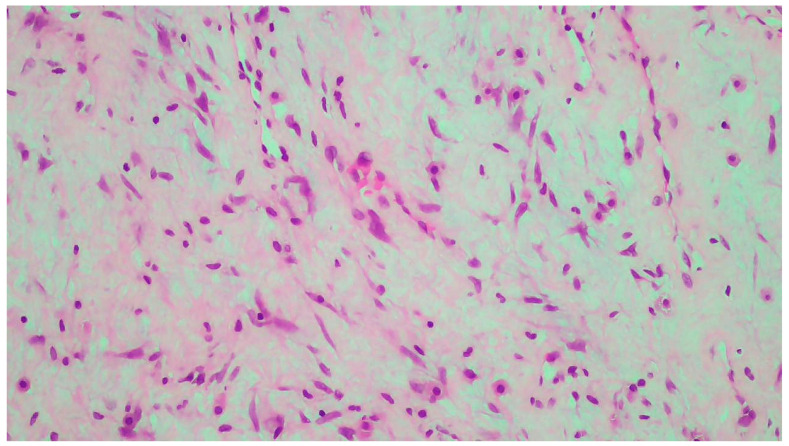
Neoplastic vessels within the myxoid and collagenous stroma.

**Figure 3:**
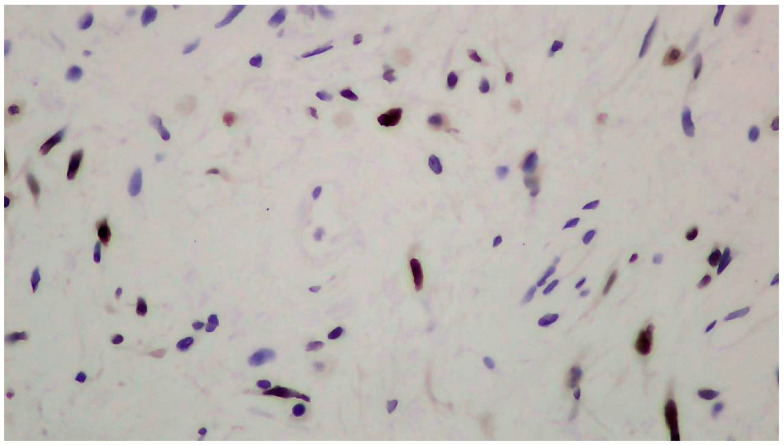
Positive immunohistochemistry for estrogen receptors (ERs+).

**Figure 4:**
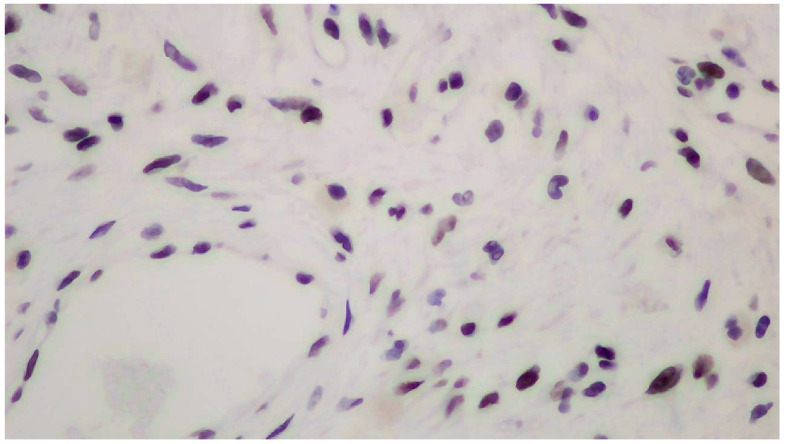
Positive immunohistochemistry for progesterone receptors (PRs+).

**Figure 5:**
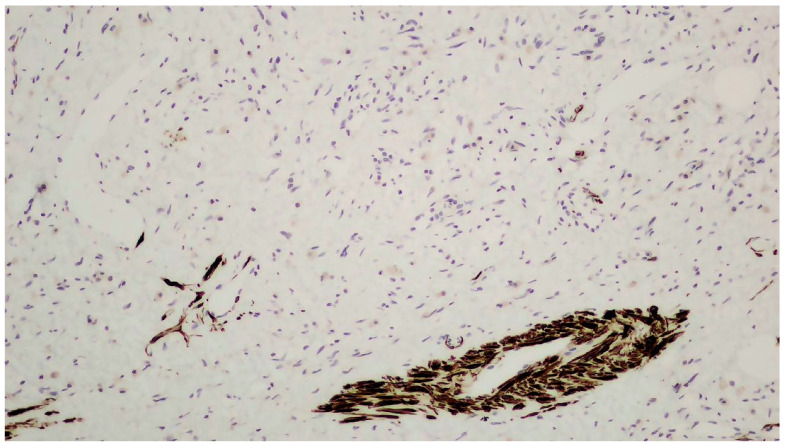
Positive immunohistochemistry for desmin.

The tumor size of this case was 6 × 6 × 3 cm and it was encapsulated. The gross appearance was consistent with a lobulated mass with fibrous bands. Each lobule consisted of myxoid and collagenous stroma with abundant thin-walled vessels and occasionally thick-walled vessels (Figure 2). Microscopic examination of the tumor showed spindle or stellate cells without atypia. Lymphocytes, plasma cells, and many mastocytes were also present. Mature adipocytes were observed in the periphery of the lobules. Immunohistochemistry was positive for estrogen receptors (ERs+) (Figure 3), progesterone receptors (PRs+) (Figure 4), and desmin (Figure 5).

The patient has been followed up for three years without detection of local recurrence. The follow-up included an ultrasound twice a year and a CT scan yearly. The patient is planned to be followed up until the fifth postoperative year.

## 3. Discussion

The clinical presentation of deep angiomyxoma is variable. Although it is a rare pelvic neoplasm, it is more common in young, premenopausal females. The age of presentation is variable as it involves children and the elderly (the minimum is 10 months and the maximum is 82 years) [8,14]. However, most patients are young females in the third to fifth decade (mean age around 35 years) and middle-aged males (mean age around 50 years) [3,9,12]. The majority of male patients present with a mass in the genitourinary region, with scrotal involvement in about half of these cases [9]. A minority of male patients report groin involvement [9]. Our patient was a 70-year-old male with an inguinal mass. Thus, our case consists of a rare presentation of deep angiomyxoma given the atypical location of the tumor in an elderly male.

The pathogenesis of deep angiomyxoma is mostly unknown. Female sex hormones (estrogen and progesterone) may play a major role in the growth of deep angiomyxoma, explaining its tendency to enlarge during pregnancy [5,11]. In addition, increased expression of high-mobility group A (HMGA) protein due to HMGA2 (12q14.3 locus, HMGIC gene) translocation leads to altered DNA transcription [5,11,15]. HMGA2 is present in approximately 60–70% of cases [4]. The origin cell of deep angiomyxoma is debated [11,12,16]. Current theories suggest origin from fibroblasts, myofibroblasts, or undifferentiated mesenchymal cells. The presence of proliferating cell nuclear antigen (PCNA) and the absence of p21 may explain the tendency for recurrence [16].

The most common complaint is painless swelling in the genital organs or the inguinal area, although atypical locations (extremities, neck, eyes, jaw, maxilla, engrafted kidney, axilla, claviculate, uterus, engrafted kidney, bladder, and greater omentum) have also been reported [9,15]. Other non-specific symptoms, such as dull pain, dyspareunia, dysuria, and constipation, often occur due to the compression of the local urogenital or gastrointestinal structures [3,9,10]. Infiltration of the adjacent visceral structures is rarely observed, which explains the absence of hematuria, hydronephrosis, and gastrointestinal bleeding [5,11,13]. The expansion of the deep angiomyxoma is gradual; thus, a long latency period without the presence of additional symptoms may be observed [12,13,16]. Consequently, the majority of patients present with large tumors (>10 cm in size) [5,11,13]. Serious complications, such as metastatic spread to the lungs and the mediastinum or pleuroperitoneal effusion with pleuroperitoneal communication, are exceedingly rare given the benign nature of this neoplasm [7,17]. Our patient presented with a slowly growing inguinal mass without gastrointestinal or urogenital complaints. The size of the neoplasm was relatively small (6 × 6 × 3 cm) without metastatic spread.

Physical examination findings are non-specific, but deep angiomyxoma is typically described as a firm and painless mass with a negative transillumination test [9]. As a result, deep angiomyxoma in a male patient is frequently misdiagnosed as an inguinal hernia, hydrocele, testicular cancer, lipoma, and epididymal cyst, misguiding the management of these patients [3,9,14]. It must be noted that the palpation of this mass only reveals the tip of the iceberg, explaining the preoperative underestimation of the tumor size [3]. The physical examination of our patient revealed an inguinal mass of 5–6 cm. The transillumination test and the Valsalva test were inconclusive.

The differential diagnosis in this case is challenging given the similar presentation of more prevalent pelvic diseases. Although there was a presumed diagnosis of inguinal hernia, this consideration was excluded by the imaging findings of a solid hyperdense mass. Hydrocele, epididymal cyst, and testicular cancer were ruled out by the absence of scrotal involvement. Consequently, there was a strong clinical suspicion of a mesenchymal tumor leading to the decision for an excisional biopsy. Finally, the microscopic appearance of the mass in combination with the immunohistochemistry findings confirmed the diagnosis of deep angiomyxoma.

Preoperative imaging is necessary for an accurate estimation of the tumor size. It may exclude other potential diagnoses and raise suspicion of soft tissue neoplasm, which can guide proper diagnostic and surgical management [2]. An ultrasound constitutes the initial imaging examination given its cost effectiveness. It typically reveals a hypoechoic and/or cystic lesion [5,10,18]. Doppler ultrasound may reveal a vascular mass [5,18]. A magnetic resonance imaging (MRI) scan with contrast is considered the most accurate imaging test, although a computed tomography (CT) scan is also a choice [5,9,10,18]. A T1-weighted MRI scan may reveal a hypointense or isointense mass compared to the muscle tissue, and T2-weighted MRI scan may reveal a hyperintense mass. A CT scan may detect a hypointense or isointense mass [5,9]. CT scan and MRI scan with contrast may identify a heterogeneously enhancing mass leading to the “swirling sign”, which is a typical feature of deep angiomyxoma [5,9,18]. However, it must be highlighted that the diagnosis of deep angiomyxoma is primarily based on pathological investigation. Hence, suspicious clinical and imaging findings must be followed by preoperative fine-needle aspiration (FNA) or core needle biopsy to make the appropriate diagnosis and surgical plan [2,19]. Unfortunately, the false-negative rate of FNA is high when the size of the tumor is lower than 10 cm [2]. In our case, a CT scan of the abdomen was ordered for the investigation of urolithiasis but also revealed a 6.5 × 5 cm hyperdense inguinal mass without cystic features. Its appearance was heterogenous.

The pathologic examination of the neoplasm after surgical excision is the gold standard for the diagnosis of deep angiomyxoma [5,9,15,18]. On gross examination, deep angiomyxoma is a gelatinous mass with a glistering and gray-white appearance. Its morphology is lobulated or polypoid with a cystic appearance, bleeding, and lack of necrosis. It is usually unencapsulated, but the presence of a capsule enables the excision of the tumor, leading to better surgical outcomes [6,10,11,20]. Large size (>10 cm) is the rule [5,11,13]. Histology reveals a hypocellular neoplasm with spindle or stellate cells in a myxoid stroma rich in vessels. The presence of thick-walled vessels is a typical feature of this neoplasm and facilitates the differential diagnosis. There is no evidence of necrosis or atypia, and mitotic activity is low or absent, which is consistent with the benign nature of deep angiomyxoma [1,2,3,4,5,6,7,8,9,10,11,12,13,14,15,16,17,18,19,20]. Immunohistochemistry is helpful for the confirmation of the diagnosis [4,5,9,15]. Vimentin, desmin, CD34, α-smooth muscle actin (α-SMA), and muscle-specific actin (MSA) tend to be positive. S100 and cytokeratin tend to be negative. Ki-67 expression is low (<1% of tumor cells) [4,5,9,15]. Vimentin, CD34, and S100 are the most useful markers [9]. Estrogen receptors (ERs) and progesterone receptors (PRs) can be positive, especially in female patients, which explains the higher prevalence, recurrence rate, and younger age of presentation among female patients [3,5,15]. ERs and PRs can be used as targets of hormone therapy. The presence of androgen receptors (ARs) has also been reported, although its significance remains unknown [9,11].

Treatment of deep angiomyxoma is primarily surgical. Despite the anatomical difficulties of the pelvis, optimal surgical removal should be widely performed to prevent local recurrence [4,5,9,15]. Notably, there was no statistically significant difference between patients with positive and negative surgical margins regarding the local recurrence rate [6,9]. However, an aggressive surgical approach for the management of a cosmetic and rarely life-threatening lesion is associated with poor cosmetic outcomes and significant disability, e.g., loss of sphincter control. Hence, surgeons should prefer more conservative approaches in difficult cases [6]. Alternative treatments include hormone therapy (second line) and embolization (third line) [4,5,6,9,15]. Hormone therapy is useful for patients with ER+/PR+ deep angiomyxoma either as adjuvant and neoadjuvant treatment or for inoperable tumors, although its efficacy remains unknown [5,9,15]. Angiography with embolization is not very successful due to the rich collateral circulation of deep angiomyxoma [5]. The use of chemotherapy and radiotherapy has been reported, but they are not recommended [5,9,15]. The treatment in our case was solely surgical due to the small size of the tumor and the easy anatomical access leading to complete resection of the mass. Although the tumor was positive for ER+/PR+ in our case, hormone therapy was not necessary given the successful surgical removal and lack of recurrence.

Follow-up is necessary for all patients given the increased probability of local recurrence, but there is no consensus regarding the timing and diagnostic testing [5,9,18]. Physical examination is not sensitive enough for the early detection of deep angiomyxoma, especially in female patients [3]. All imaging modalities (ultrasound, CT, MRI) have been proposed as screening tools depending on the patient and the location of the primary tumor [9,18,20]. In our study, a combination of ultrasound and CT scan was selected as the follow-up method, without the detection of recurrence during the 3-year follow-up period.

## 4. Conclusions

Deep angiomyxomas are rare neoplasms, especially in male patients. The diagnosis is challenging due to non-specific clinical findings, but it should be considered in patients with a large pelvic mass. The differential diagnosis is broad and includes more common diseases, such as abdominal hernia, hydrocele, epididymal cyst, testicular mass, and soft tissue tumors, e.g., lipoma. Pre-operative diagnosis with imaging (ultrasound, CT, MRI) and biopsy is essential in managing these cases. Suspicious findings include a hypoechoic or cystic appearance with increased vasculature on ultrasound and a hypointense or isointense mass on CT. MRI is the best imaging study; suggestive findings include a hypointense or isointense mass (T1 sequence) and a hyperintense mass (T2 sequence). However, the biopsy is the most accurate diagnostic test. The diagnosis of deep angiomyxoma should always be considered in patients with an inguinal mass to avoid delayed treatment, incomplete surgical excision, and violation of adjacent organs.

## Data Availability

The data used in the current study are available from the corresponding author upon reasonable request.
